# Nanostructured Nickel Aluminate as a Key Intermediate for the Production of Highly Dispersed and Stable Nickel Nanoparticles Supported within Mesoporous Alumina for Dry Reforming of Methane

**DOI:** 10.3390/molecules24224107

**Published:** 2019-11-14

**Authors:** Leila Karam, Julien Reboul, Nissrine El Hassan, Jaysen Nelayah, Pascale Massiani

**Affiliations:** 1 Sorbonne Université, Campus UPMC, CNRS UMR-7197, Laboratoire de Réactivité de Surface, 4 Place Jussieu, F-75005 Paris, France; laylakaram_22@hotmail.com (L.K.); julien.reboul@upmc.fr (J.R.); 2 Department of Chemical Engineering, Faculty of Engineering, University of Balamand, P.O. Box 33, Amioun, 33 El Koura, Lebanon; nissrine.hassan@balamand.edu.lb; 3 Laboratoire Matériaux et Phénomènes Quantiques, Université de Paris, CNRS, F-75013 Paris, France; jaysen.nelayah@univ-paris-diderot.fr

**Keywords:** nickel aluminate, mesoporous alumina, heterogenous catalysis, MOF, HAADF, dry reforming of methane (DRM)

## Abstract

Two routes of preparation of mesoporous Ni-alumina materials favoring the intermediate formation of nanostructured nickel-aluminate are presented. The first one involves an aluminum containing MOF precursor used as sacrificial template to deposit nickel while the second is based on a one-pot synthesis combined to an EISA method. As shown by a set of complementary techniques, the nickel-aluminate nanospecies formed after calcination are homogeneously distributed within the developed mesoporous alumina matrices whose porous characteristics vary depending on the preparation method. A special attention is paid to electron-microscopy observations using especially STEM imaging with high chemical sensitivity and EDS elemental mapping modes that help visualizing the extremely high nickel dispersion and highlight the strong metal anchoring to the support that persists after reduction. This leads to active nickel nanoparticles particularly stable in the reaction of dry reforming of methane.

## 1. Introduction

Dry reforming of methane (DRM), a reaction that converts CH_4_ and CO_2_ into a mixture of CO and H_2_ (synthesis gas), is gaining significant attention in the research sector. This process uses CH_4_ and CO_2_ (greenhouse gases) extracted from natural reserves or produced from renewable sources such as municipal solid wastes, and converts them into valuable syngas that is a feedstock for the production of synthetic fuels and of chemical intermediates in petrochemical industries. As such, DRM serves as a promising alternative of the existing depleting fossil fuels and minimizes global warming [1,2,3]. However, the main problem that plagues this reaction from reaching the industrial scale is the challenges faced upon constructing an active and stable catalyst. Despite their good performance, catalysts involving noble metals such as Pt, Pd, Ru, Ir or Rh are avoided because they are expensive and scarcely available [4,5]. As an alternative, Ni-based catalysts are widely studied due to their lower price, high abundance, and good activity that make them industrially economical [6,7]. The drawback of such catalysts however is their tendency to deactivate at the high reaction temperatures required by the reaction that favor sintering of the supported Ni active phase and formation of coke deposition. Efforts have been put to strengthen the stability of such materials by several means, such as the addition of a second metal or promoter [8,9], altering the type of support used [10] or enhancing the interaction between Ni and its carrier [11].

**Scheme 1:**
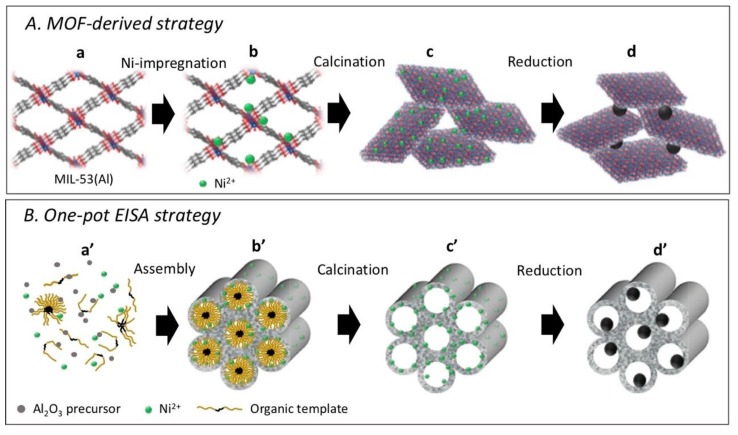
Schematic representation of the (**A**) MOF-derived and (**B**) one-pot EISA synthesis routes consisting of four main steps: in (**A**), the parent MIL-53(Al) (step a) is impregnated with a Ni^2+^ precursor to obtain a nanostructured hybrid material where Ni^2+^ (in green) and Al^3+^ (in the nods, in red) are in close proximity (step b), then calcination leads to a high surface area Ni-Al_2_O_3_ mixed oxide (step c) that is finally reduced under H_2_ to give the porous Ni^0^-Al_2_O_3_ catalyst (step d); in (**B**), the one-pot EISA route consists of mixing all materials components (Ni^2+^ and Al^3+^ precursors, organic structuring agent and solvent) in the synthesis gel (step a’), leading after evaporation to an hexagonally assembled hybrid Ni^2+^/Al^3+^ oxide material (step b’) that is next calcined to liberate the porosity (step c’) and is finally reduced to form the well-dispersed Ni^0^ active nanoparticles (step d’).

Regarding the latter strategy, a way to improve Ni-support interaction is to intimately mix nickel cations within the metal oxide support from the first steps of preparation of the materials, namely before the calcination step leading to the formation of the targeted alumina. To this end, we recently developed two distinct synthesis routes of mesoporous nickel alumina DRM catalysts. In the first approach (Scheme 1A), a metal-organic-framework (MOF) of high surface area was used as sacrificial precursor (step a); it contained aluminum-hydroxyl nods bridged by organic linkers able to act as anchorage sites for nickel ions after impregnation (step b); after decomposing the linkers by thermal oxidation (step c) and then reducing the sample (step d), a porous nickel alumina catalyst with very small and highly stable Ni nanoparticles dispersed on thin γ-Al_2_O_3_ layers was obtained [12]. The second approach (Scheme 1B) consisted of the direct addition of nickel within the synthesis medium of the alumina matrix (one-pot procedure, step a’) combined to an evaporation-induced self-assembly (EISA) method (step b’), leading after thermal oxidative decomposition of the organic structuring agent (assemblies of block copolymers) to a hexagonally structured mesoporous Ni-alumina material with Ni^2+^ species occluded inside the Al_2_O_3_ walls (step c’) and remaining strongly attached to the support after reduction (step d’) [13]. With both strategies, a key-step for obtaining highly stable metal nanoparticles in the reduced catalysts seemed to rely on the intermediate formation, after the thermal removal of the organic structuring agents or linkers present in the fresh materials, of nickel aluminate (NiAl_2_O_4_) nanodomains inserted within the thin inorganic high specific surface alumina walls. The high specific area of the materials also played an important role by improving the accessibility of the reactants to the nickel active sites.

However, the beneficial effect of such mixed oxide phase in the context of Ni^0^-based catalysts for DRM is still subject of debate. Indeed, in agreement with our observations, some studies report that the intermediate nickel insertion in the form of NiAl_2_O_4_ strengthens the metal-support interaction in the reduced catalyst and contributes to a decrease of Ni^0^ particles sizes compared to those issued from the reduction of bulk NiO crystals located on the alumina surface [14,15,16,17]. Contrarily, other researchers consider that NiAl_2_O_4_ acts as an inert phase in the DRM reaction, making also more difficult the reduction of Ni^2+^ into active Ni^0^ and obstructing the access to the active sites [14,18,19]. In this context, the present contribution aims to highlight the positive effect of nickel aluminate species formed within high surface area mesoporous nickel-alumina materials obtained according to the routes illustrated in Scheme 1. To this end, two samples with 5 wt% Ni were prepared, according to conditions detailed in Section 3, and denominated Ni_5wt%_-Al_2_O_3-MOF_ and Ni_5wt%_-Al_2_O_3-EISA_ for the MOF-derived and one-pot EISA strategies, respectively. Please note that Section 3, entitled “Materials and Methodologies”, also introduces the techniques of characterizations and conditions used for the catalytic tests. As a whole, this work targets a better understanding of the enhanced interactions between Ni and Al_2_O_3_ inducing high dispersion and good catalytic performance.

## 2. Results and Discussion

### 2.1. Mesoporous Organizations and Morphologies

**Figure 1:**
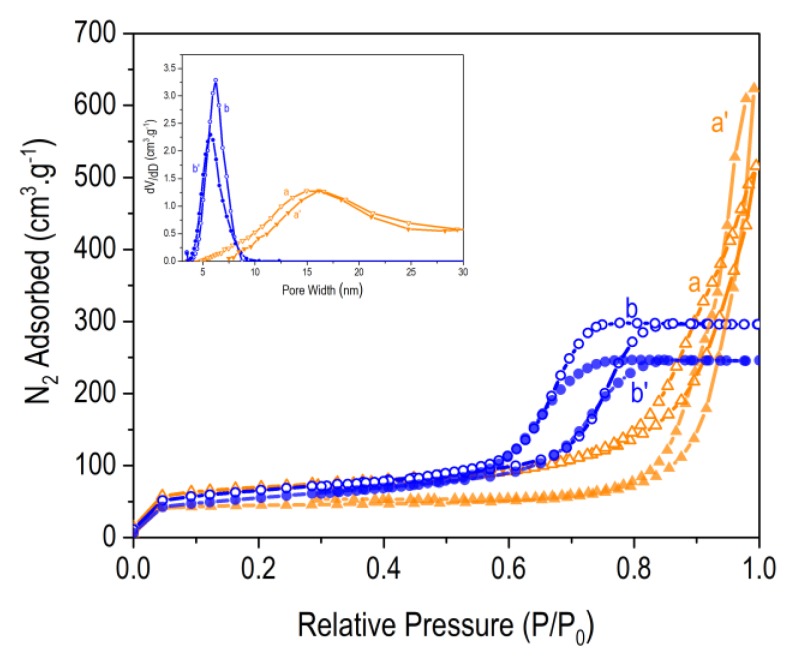
N_2_ physisorption isotherms and pores sizes distribution (in inset) after degassing at 250 °C of the (**a**,**b**) calcined and (**a’**,**b’**) reduced materials: (**a**,**a’**) Ni_5wt%_-Al_2_O_3-MOF_ and (**b**,**b’**) Ni_5wt%-_Al_2_O_3-EISA_.

The porous characteristics of the freshly prepared (i.e., calcined) materials were first analyzed by N_2_ adsorption-desorption, a technique that informs on pores organization and surface area in the materials (see details on measured parameters in Section 3). For both Ni_5wt%_-Al_2_O_3-MOF_ and Ni_5wt%_-Al_2_O_3-EISA_ materials, the isotherms are of type IV and show a hysteresis loop typical of the presence of mesopores (Figure 1). However, the loops are quite distinct depending on the synthesis procedure, revealing different textural properties. For Ni_5wt%_-Al_2_O_3-MOF_ (Figure 1a), the hysteresis loop is of type H3, showing (i) a progressive increase of the adsorbed N_2_ volume at P/P_0_ higher than 0.75, (ii) a slit-type hysteresis spread over P/P_0_ values between 0.75 to 1 and (iii) an absence of plateau at P/P_0_ between 0.9 and 1. This is characteristic of a layered material containing large mesopores with a relatively polydisperse size distribution [15]. Such profile and the resulting BET specific surface of 240 m^2^·g^−1^ (S.A., Table 1) contrast with the conventional type I isotherms and very high surface area (often above 500 m^2^·g^−1^) traditionally observed for organized high surface area microporous MOF systems and specifically for MIL-53(Al) [16]. The presence of large mesopores in Ni_5wt%_-Al_2_O_3-MOF_ is due to the organic linker degradation and simultaneous inorganic (alumina) oxide formation that takes place during calcination of the impregnated MOF-based material at 500 °C (Scheme 1A, b,c).

The shape of the isotherm drastically differs for the Ni_5wt%_-Al_2_O_3-EISA_ material that was prepared in presence of amphiphilic block copolymers assemblies as structuring agent (see details on the one-pot EISA method in Section 3). Indeed, this sample exhibits a H1 hysteresis loop (between P/P_0_ = 0.57 and 0.80), indicative of uniform and well-organized cylindrical mesopores with an average pore diameter (Ø) and a pore volume (V_tot_) smaller than in Ni_5wt%_-Al_2_O_3-MOF_ (Table 1). Nevertheless, the specific surface of Ni_5wt%_-Al_2_O_3-EISA_ ((S.A = 230 m^2^·g^−1^, Table 1) is close to that of Ni_5wt%_-Al_2_O_3-MOF_. For both samples, the porous characteristics are preserved after reduction at 800 °C (Figure 1a’,b’) with however a small decrease of the specific surfaces and pore volumes easily explained by the high temperature treatment provoking some structural shrinkage (Table 1).

**Figure 2:**
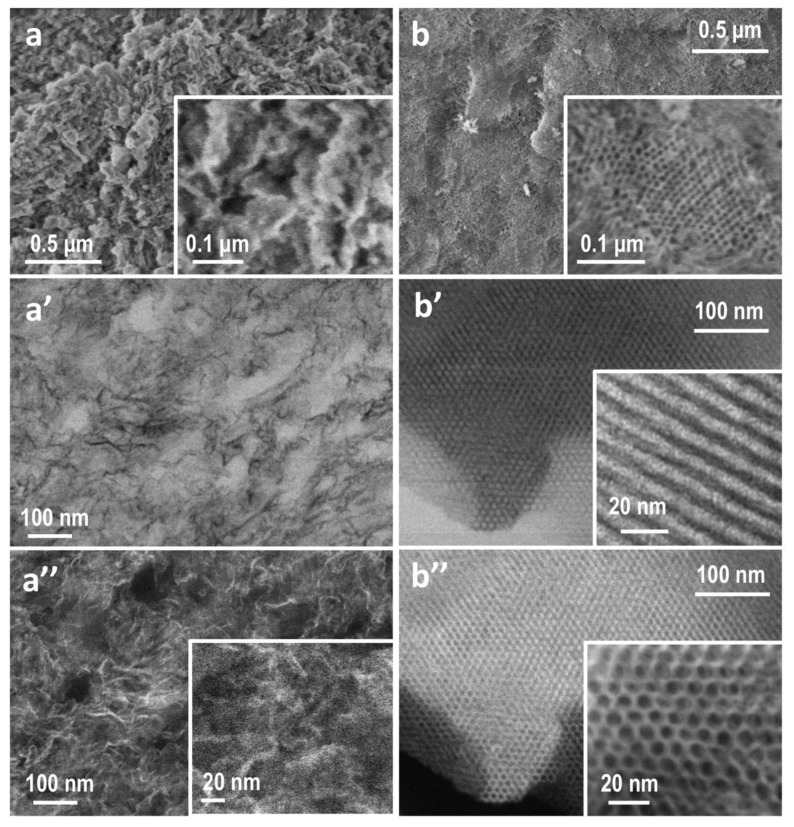
(**a**,**b**) SEM, (**a’**,**b’**) STEM-BF and (**a”**,**b”**) STEM-HAADF images of (**a**,**a’**,**a”**) Ni_5wt%_-Al_2_O_3-MOF_ and (**b**,**b’**,**b”**) Ni_5wt%_-Al_2_O_3-EISA_ (calcined forms).

Textural differences between the calcined Ni_5wt%_-Al_2_O_3-MOF_ and Ni_5wt%_-Al_2_O_3-EISA_ samples were also confirmed by electron microcopy studies carried out in both scanning (Figure 2a,b) and transmission (Figure 2a’,b’) modes. For the latter, the observations were performed on ultra-thin microtomic sections in order to allow a correct identification of the porosities and of the nickel species expected to be present within the materials. In scanning mode, Ni_5wt%_-Al_2_O_3-MOF_ depicts slightly elongated aggregated nanograins with uniform sizes of approximately 20 nm in length (Figure 2a). STEM bright-field (STEM-BF) imaging shows that these grains are in fact constituted of fused ill-defined and defective alumina flakes composed of several randomly interwoven nanosheets (Figure 2a’). This morphology fully agrees with the layered aspect deduced above from the isotherm. With respect to Ni_5wt%_-Al_2_O_3-EISA_, the grains observed at low magnification (SEM, Figure 2b) seem to be bigger than in Ni_5wt%_-Al_2_O_3-MOF_, but a closer examination shows that they are constituted of interconnected aggregates of small crystals containing ordered 2D p6mm hexagonal mesoporous channels. This is revealed by the pores’ apertures appearing on their surface (inset in Figure 2b) and by the very well resolved channels visible on STEM images visualizing alumina grains positioned with their channels either parallel (Figure 2b’) or perpendicular (inset in Figure 2b’) to the electronic beam. On the HR-TEM pictures; however, and despite the high resolution accessible on the used microscopes, nickel-based nanoparticles were never detected, neither in the pores nor in the alumina walls, and this was also the case in the Ni_5wt%_-Al_2_O_3-MOF_ nanosheets. Since nickel is known to be present in the two samples (because it was added during their synthesis), additional observations were made using the high angular dark field (STEM-HAADF) method which principle is to discriminate compounds with different atomic numbers (weights), thus helping to differentiate between the Ni (Z = 28) and Al (Z = 13) elements. The images thus recorded on the same zones as above (Figure 2a”,b”) strongly resemble those obtained in STEM-BF mode (except for the reverse contrast due the specificity of each technique). Notably, they do not show any appearance of white spot that would have revealed local Ni enrichment or presence of NiO nanoparticles as is traditionally observed for such types of materials. This reveals an extremely high nickel dispersion within the alumina matrices, whatever the sample.

### 2.2. Evidence of a Nickel Aluminate Spinel Phase in the Calcined Materials

**Figure 3:**
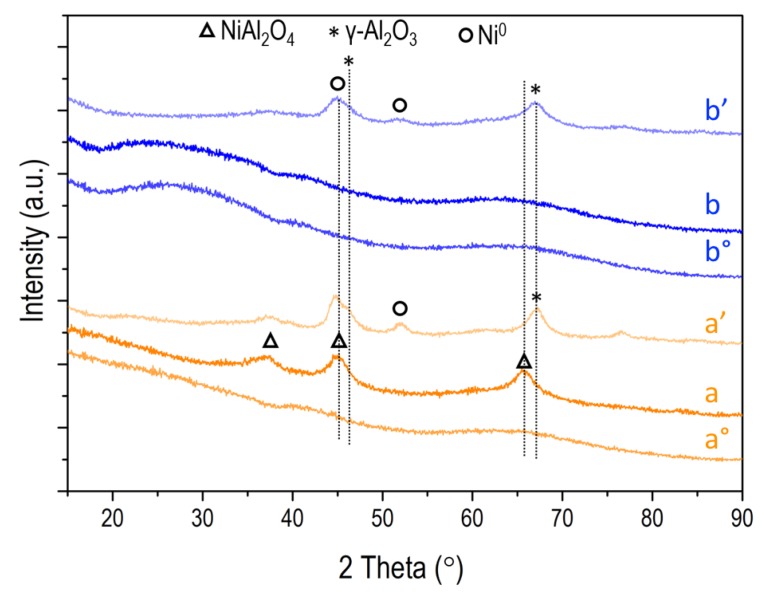
X-ray diffractograms of the prepared (**a°**,**b°**,**a**,**b**) calcined and (**a’**,**b’**) reduced materials: (**a°**) Al_2_O_3-MOF_, (**b°**) Al_2_O_3-EISA_, (**a**,**a’**), Ni_5wt%_-Al_2_O_3-MOF_ and (**b**,**b’**) Ni_5wt%_-Al_2_O_3-EISA_.

X-ray diffraction was carried out to obtain information on crystalline phases present in the materials. The patterns of calcined Ni_5wt%_-Al_2_O_3-MOF_ and Ni_5wt%_-Al_2_O_3-EISA_ are shown in Figure 3 (curves a and b, respectively) together with those of their Ni-free Al_2_O_3-MOF_ and Al_2_O_3-EISA_ analogues synthesized under the same conditions except for the absence of nickel (curves a° and b°, respectively). Both alumina supports (without Ni) displayed only very broad and weak peaks (2θ ranges 30–40° and 60–70°) assignable to amorphous alumina (Figure 3a°–b°). In the case of Ni_5wt%_-Al_2_O_3-EISA_, the addition of nickel did not affect the diffractogram (Figure 3b), indicating that the formed Ni-based species were amorphous and/or highly dispersed. Together with above STEM data, this reveals that crystalline nickel nano-species, if present, have sizes below the size detection limit of the used equipment. In contrast, nickel addition provoked the appearance of few weak peaks at 2θ of 37.0°, 44.9° and 65.5° when following the MOF-based synthesis route (Figure 3a). These positions slightly differ from the ones expected for the face centered cubic cell of NiO (2θ at 37.2°, 43.1° and 62.8° for planes (111), (200) and (220), respectively, JCPDS: 01-071-4750) and rather correspond to a nickel aluminate NiAl_2_O_4_ spinel phase (JCPDS: 001-1299).

Hence, the calcination step made to remove the organic linkers from the parent MOF material simultaneously induced a local structuring between the impregnated nickel ions and the aluminum hydroxyl nods brought close to each other within the MOF upon impregnation. Nickel aluminate is a crystalline spinel phase that belongs to a cubic system with Fd-3m space group and its structure consists of an ensemble of tetrahedral coordination occupied with bivalent Ni^2+^ and octahedral coordination filled with trivalent Al^3+^ cations [17]. The formation of this phase relies on the diffusion of Ni^2+^ and Al^3+^ at the interface between the metal oxides Al_2_O_3_ and the NiO formed at the early stages of the thermal treatment. This cation diffusion is known to require a high energy input and NiAl_2_O_4_ is hence commonly obtained at temperatures above 550 °C [20]. The presence of such phase in Ni_5wt%_-Al_2_O_3-MOF_ although it was calcined only at 500 °C (the temperature used to decompose the organic linker) is likely due to the close proximity between Ni^2+^ and the preformed AlO_6_ octahedra composing the inorganic chains of the MOF framework that facilitates the cations diffusion and thereby decreases the energy of the spinel phase formation. The mean particle size of the crystalline NiAl_2_O_4_ domains estimated by Scherer equation in this MOF-based sample is around 4.6 nm, a little bit smaller than the 5.1 nm NiAl_2_O_4_ crystallites reported earlier by Fan X. et al. in a study of a mesoporous nickel-alumina material with 5 wt% Ni [21]. At this point, it can be added that such sizes keep rather small for species that contain both nickel and aluminum atoms, explaining why the Ni-based nanospecies could not be detected by TEM in the calcined materials, neither in Ni_5wt%_-Al_2_O_3-MOF_ nor in Ni_5wt%_-Al_2_O_3-EISA_. Nevertheless, the presence of nickel and its homogeneous distribution throughout the alumina matrices were totally confirmed by local TEM/EDS analyses in both calcined samples (data not detailed).

**Figure 4:**
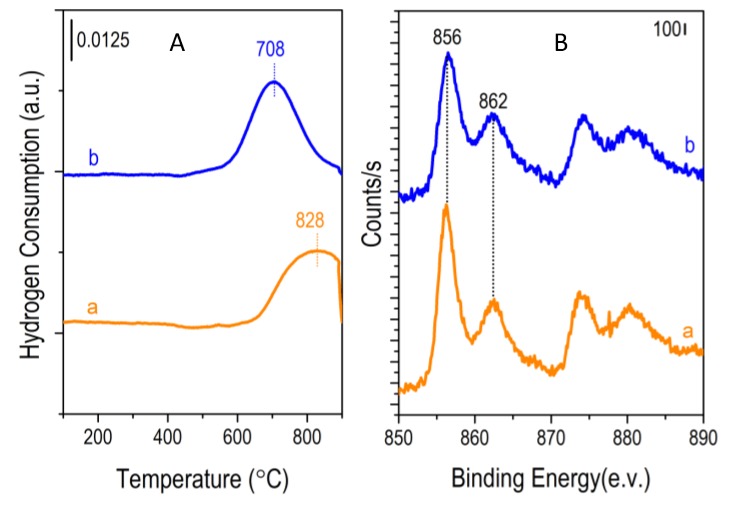
(**A**) TPR profiles and (**B**) XPS spectra of samples (a) Ni_5wt%_-Al_2_O_3-MOF_ and (b) Ni_5wt%_-Al_2_O_3-EISA_ (calcined forms).

The presence of nickel and its content were also validated by temperature programed reduction (TPR) experiments during which oxidized nickel (Ni^2+^ ions or NiO) was transformed to Ni^0^ (the active state for DRM). For both Ni_5wt%_-Al_2_O_3-MOF_ and Ni_5wt%_-Al_2_O_3-EISA_ samples, the TPR profiles (Figure 4A) did not present any peak at temperatures below 500 °C where the reduction of bulk NiO deposited on alumina surfaces and weakly interacting with them is usually expected [11]. They rather exhibited a single and symmetric reduction peak at elevated temperature (above 700 °C) referring to the presence of nickel strongly interacting with the alumina support. The related H_2_ uptake was systematically close to the theoretical value of 860 μmol·g^−1^ expected for 5 wt% Ni assuming a consumption of one H_2_ molecule per divalent nickel atom (Ni^2+^).

The observed high reduction temperature fits well with the reduction of Ni^2+^ ions homogeneously embedded into Al_2_O_3_ lattices and forming nanocrystalline NiAl_2_O_4_ spinel domains [18,21,22] as depicted above by XRD. The slightly lower reduction temperature for Ni_5wt%_-Al_2_O_3-EISA_ compared to Ni_5wt%_-Al_2_O_3-MOF_, together with the absence of XRD peak for the former sample, might indicate the formation of less structured or smaller mixed oxide species in the alumina walls of the hexagonally organized material, with slightly weakened nickel-alumina interaction. This might result from a higher nickel dispersion in the aqueous one-pot EISA synthesis medium than in the impregnated MOF sample, leading to especially well distributed and tiny (hence less structured) Ni-based species after calcination. Moreover, a further evidence of the formation of nickel aluminate spinel in the nickel alumina mixed layers or walls of the prepared materials is given by XPS spectra that show a main Ni2p_3/2_ peak centered at 856 eV accompanied by a shake-up satellite around 862 eV (Figure 4B). Such positions confirm the formation of an intimate nickel-alumina oxide solution and exclude the presence of pure NiO which binding energy would be expected at 853 eV [23]. Besides, the quantitative analysis of XPS data confirmed as well the nickel content in both samples.

### 2.3. Resulting Ni^0^ Dispersion and Catalytic Performance of the Reduced Catalysts

**Figure 5:**
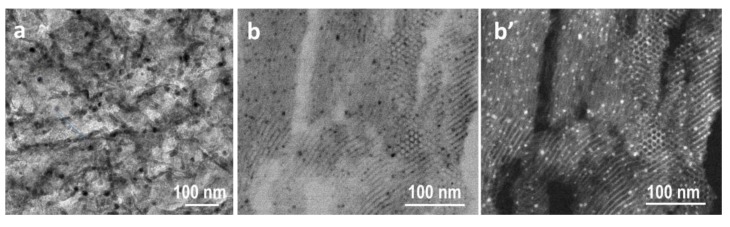
Micrographs of reduced (**a**) Ni_5wt%_-Al_2_O_3-MOF_ and (b-b’) reduced Ni_5wt%_-Al_2_O_3-EISA_ catalysts; images recorded in (**a**) HR-TEM, (**b**) STEM-BF and (**b’**) STEM-HAADF modes.

Nickel, which was not visible in TEM images of calcined materials, became apparent after reduction, as visualized by the small dark spots contrasting with the grey color of the support in the TEM images of both reduced Ni_5wt%_-Al_2_O_3-MOF_ (Figure 5a) and Ni_5wt%_-Al_2_O_3-EISA_ (Figure 5b) catalysts. For Ni_5wt%_-Al_2_O_3-EISA_, the Ni^0^ nanoparticles were very small, being hence better seen in HAADF mode (Figure 5b’) than in more conventional bright field mode (Figure 5b). The histograms of particle sizes established from high resolution TEM images after measuring the sizes of at least 500 nanoparticles led to mean Ni^0^ nanoparticles sizes of 6.8 and 7 nm in Ni_5wt%_-Al_2_O_3-MOF_ and Ni_5wt%_-Al_2_O_3-EISA_, respectively.

The presence of Ni^0^ nanoparticles in both reduced Ni_5wt%_-Al_2_O_3-EISA_ (Figure 3a’) and Ni_5wt%_-Al_2_O_3-MOF_ (Figure 3b’) was also demonstrated by X-ray diffraction. Thus, the patterns depicted new diffraction peaks centered at 2θ = 44.5°, 51.9° and 76°, assignable to the (111), (200) and (220) planes of crystalline Ni^0^ (JCPDS: 04-850). Additional bands attributable to a structured alumina-based phase appeared as well, with positions characteristic of pure γ-Al_2_O_3_ (identified by an asterisk), and they substituted those of crystalline nickel aluminate present before reduction. Put together, these observations can be interpreted by a Ni^2+^ extraction from the oxide matrix towards the pore surface during nickel reduction followed by an aggregation in the form of Ni^0^ nanoparticles. The mean Ni^0^ nanoparticle sizes estimated by applying the Scherrer equation to the Ni^0^ reflection at 2θ = 51.9° (chosen because of not overlapping with any other signal) are between 5 and 6 nm in both reduced Ni_5wt%_-Al_2_O_3-MOF_ and Ni_5wt%_-Al_2_O_3-EISA_, which is in the range—even though slightly below - the above values established from HR-TEM observations. This may be due to the uneasy identification of very small nanoparticles by microscopy.

**Figure 6:**
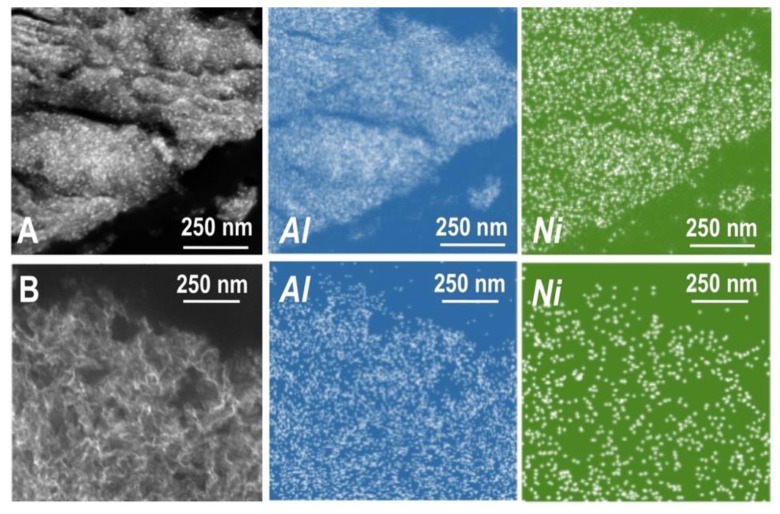
STEM (**left images**) and EDS mappings of Al (**middle**) and Ni (**right**) elements for representative grains of reduced (**A**) Ni_5wt%_-Al_2_O_3-MOF_ and (**B**) Ni_5wt%_-Al_2_O_3-EISA._

Importantly, all detected nickel nanoparticles appear homogeneously distributed over the alumina matrices (Figure 5) and this is further illustrated by STEM/EDS mappings that show Ni and Al elemental superimpositions in both catalysts (Figure 6). The alumina grains still appear as interwoven nanosheets in Ni_5wt%_-Al_2_O_3-MOF_ (Figure 5a) and as hexagonally arranged mesoporous parallel channels in Ni_5wt%_-Al_2_O_3-EISA_ (Figure 5b). This preservation of morphologies after reduction is also attested by textural properties, the N_2_ physisorption isotherm still showing type IV isotherms with a slit-like hysteresis typical of a layered material composed of poorly organized interlayered pores for reduced Ni_5wt%_-Al_2_O_3-MOF_ (Figure 1a’) and a well-defined H1 hysteresis loop for reduced Ni_5wt%_-Al_2_O_3-EISA_ resembling that before reduction (Figure 1b’). The small decrease in surface area reveals however some structural shrinkage due to the harsh high temperature reduction treatment (Table 1).

The performance of the reduced catalysts in dry reforming of methane carried out at atmospheric pressure, 650 °C and under a high space velocity (GHSV = 72 L·g^−1^·h^−1^) are given in Table 1 as reactants conversions (CH_4_ and CO_2_) and H_2_/CO products ratio. With both catalysts, conversions were remarkable, close to thermodynamic equilibrium, and higher by approximately 3 times than on a reference Ni-alumina catalyst prepared by a conventional wetness impregnation method and tested for comparison in the same conditions [12]. This is not only due to the excellent Ni^0^ dispersion providing a high number of active sites within the mesoporous Ni-alumina-based catalysts (as also confirmed by H_2_-TPD, data not shown) but also to the high accessibility to the active sites provided by their high specific surfaces. Nevertheless, the slightly higher conversions of CO_2_ compared to those of CH_4_ and the concomitant H_2_:CO products ratios slightly above 1 reveal the occurrence of two side reactions frequently encountered at such high reaction temperatures, namely reverse water gas shift (RWGS, consuming CO_2_ and H_2_ to produce CO and H_2_O) and methane decomposition (producing 2H_2_ per CH_4_ converted). Their extent was however limited and the catalysts are especially stable, with no loss of activity and product selectivity when the catalytic tests are prolonged during 13 h (data not detailed).

To sum up, and considering all these data, it can be proposed that the improved stability of the Ni^2+^ ions isolated within nickel aluminate nanodomains in the calcined mesoporous materials with high specific surface slows down their extraction from the support during the reducing step, thus contributing to the formation of numerous tiny Ni^0^ nuclei that grow on the alumina surface at a limited extent to give small nanoparticles instead of a few nuclei evolving as large metal particles. The improved stability of these highly dispersed Ni^0^ nanoparticles should rely on the occurrence of a certain amount of remaining nickel aluminate or related phases at the interface between the metal nanoparticle and the oxide support affording a particularly strong chemical interaction and hence acting as a “glue” between the particle and its alumina support. Indeed NiAl_2_O_4_ can be altered when Ni^2+^ partially adopts octahedral site while the tetrahedral site hosts Al^3+^ together with Ni^2+^ ions resulting in compounds of inverse spinel structure known as Ni_1−x_[NixAl_2−x_]O_4_ where 0 < x < 1. This structure flexibility at the metal-support interface may enhance the interaction between Ni and the support, restrict the aggregation of metallic Ni sites, and increase their dispersion as mentioned in previous works [21,24,25]. In the conditions of DRM reaction, this inhibits not only nickel sintering but also the potential formation of carbon nanotubes which growth necessitates the presence of weakly attached reduced nickel particles able to move far away from the support itself by being embedded at the tip of the nanotubes while these nanotubes form [26]. We can also speculate that the nickel aluminate domains inserted in the nanostructured calcined intermediates as well as their resulting Ni^0^ active nanoparticles are all located on (or close to) the high area mesoporous surfaces, being hence easily accessible to reactants during the reaction.

## 3. Materials and Methods

For the MOF-based preparation, a parent Al containing MIL-53(Al) sample (MOF family) was chosen and synthetized according to the method of Isaeva V. et al. [27]. Briefly, 1.21 g of AlCl_3_·6H_2_O and 0.42 g of benzene-1,4-di-carboxylic acid were dissolved in a solution made of 3 mL water and 5 mL dimethylformamide (DMF). The mixture was transferred into a 50 mL tubular reactor and heated in a microwave oven for 30 min at 125 °C and a power of 200 W. The formed crystalline product was washed 6 times with 10 mL of DMF followed by rinsing with 10 mL of deionized water, and recovered each time by centrifugation. After drying for 24 h at 70 °C, the sample was calcined in air at 220 °C for 72 h to remove adsorbates from the pores, then impregnated with a Ni(NO_3_)_2_·6H_2_O aqueous solution as to obtain a final nickel content of 5 wt%, and finally heated in a muffle furnace (thin bed conditions) at a rate of 0.5 °C·min^−1^ till keeping the temperature at 500 °C for 5 h (sample Ni_5wt%_-Al_2_O_3-MOF_). For comparison, a reference Ni-free alumina sample was similarly synthesized except for the impregnation step (sample Al_2_O_3-MOF_).

The other organized mesoporous catalyst was synthesized in presence of nickel ions using a one-pot evaporation induced self-assembly (EISA) method [13,28,29] as follows. 1 g of P123 Pluronic triblock copolymer was dissolved in 20 mL of absolute ethanol at ambient temperature (25 °C) under intense stirring, then 1.6 mL of HNO_3_, 5 mmol of Al[OCH(CH_3_)_2_]_3_ and 5 mmol of Ni(NO_3_)_2_·6H_2_O were added still under stirring. The obtained mixture was covered with a polyethylene film (PE) and stirred overnight to reach a complete dissolution of the chemicals. Next, the solution was dried for 48 h at 60 °C to slowly evaporate ethanol and HNO_3_. The resulted Xerogels were finally calcined as above at 500 °C under static air for 5 h (sample Ni_5wt%_-Al_2_O_3-EISA_). Again, a reference Ni-free alumina material was prepared following the same procedure but without Ni addition (sample Al_2_O_3-EISA_).

N_2_ adsorption-desorption isotherms were recorded at −196 °C on an ASAP 2020 Micromeritics apparatus. After insertion in the analysis tube, the sample was degassed under vacuum for 2 h at 250 °C then placed at liquid nitrogen temperature before plotting the N_2_-isotherm. Specific surfaces were calculated from BET equation at a relative pressure between 0.05 and 0.25. The corresponding single point pore volumes were obtained from the sorption branch at a relative pressure of 0.99. Pore size distributions were calculated based on the BJH formula which was applied on the desorption branch.

Scanning Electron Microscopy (SEM) images were registered in mixed mode (70% secondary electrons and 30% retro-diffused signals) on a Hitachi SU-70 SEM-FEG microscope with an electron acceleration tension of 7 kV. High-Resolution transmission electron microscopy observations were done on 50–70 nm sections of the samples prepared by microtomic cutting as detailed elsewhere [12,13]. The TEM micrographs and EDS elemental mappings were registered on a JEOL-JEM 2020 electron microscope with LaB6 gun. The STEM images were acquired on a JEOL ARM 200F instrument (JEOL Ltd., Tokyo, Japan) operated at 200 kV as well, but equipped with a FEG source and an aberration corrector of the objective lens. The probe setting in STEM mode was 8c. The condenser lens aperture size and the camera lens used were 30 μm and 6 cm respectively. With these settings, the corresponding convergence semiangle was 12 mrad, and the inner and outer collection semiangles on the STEM-HAADF detector were 90 and 390 mrad, respectively. Average sizes of nickel particles, when visible, were estimated using the ImageJ software and taking at least 500 particles into consideration.

Powder X-ray diffraction (XRD) measurements were performed in the 2θ range 20–90°on a PANalytical XPert3 diffractometer using a CuKα radiation (λ = 1.5405 nm), a voltage of 30 Kv, a current of 10 mA and a time step of 2 s. Known ICDD powder XRD files were used to identify crystalline phases and mean sizes of crystalline domains (nanoparticles) were calculated using the Scherer’s equation Dhkl = Kλ/βcosθ, where K is the shape factor of the average spherical crystalline, λ is the wavelength (1.5405 nm for CuKα), β is the full width at half maximum (FWHM) and θ is the peak position.

Hydrogen temperature programmed reduction (H_2_-TPR) was operated on an Autochem 2920 Micromeritics unit. A certain amount (≈70 mg) of the calcined sample was put in a U-shaped quartz reactor and heated (10 °C·min^−1^) from ambient temperature up to 900 °C under 25 mL·min^−1^ of 5 vol% H_2_/Ar. The amount of H_2_ consumed was determined by TCD after passing the effluent gas in a tube placed in a bath composed of ice and NaCl to trap any possible water formed during reaction (H_2_(g)+NiO(s)→ Ni^0^(s)+H_2_O(g)) and thus assert on the sole detection of H_2_ consumption.

XPS spectra were obtained on an Omicron (ESCA+) X-ray photoelectron spectrometer with an Al Kα (hν = 1486.6 eV) X-ray source with a 300 W electron beam power. The sample was evacuated under a vacuum of less than 10^−10^ mbar, then a monochromatic X-ray (Al Kα) irradiated the sample and excited the electrons. Surface compositions were determined by attributing the observed energy peaks to a specific element and quantifying their area using the Casa XPS software.

Stability tests during dry methane reforming reaction were conducted in a fixed bed reactor after in situ reduction of the calcined material at 800 °C for 2 h (heating rate 5 °C·min^−1^) under 30 mL·min^−1^ of 5 vol% H_2_/Ar. The tests were carried out at 650 °C and atmospheric pressure for 10 h under a flowing reactant gas composed of an equimolar amount of CH_4_ and CO_2_ diluted in Ar (atomic ratio 5/5/90). The hourly space velocity (GHSV) was chosen equal to 72 L·g^−1^·h^−1^ from previous measurements [17,20]. Catalytic performance was established from the gas composition at the exit of the reactor monitored with an INFICON micro GC along with a TCD and two parallel channels (Molecular sieve and Plot U). The data are expressed as reactants (CH_4_ and CO_2_) conversions and products (H_2_ and CO) mole ratios.

## 4. Conclusions

In view of this work, key features for preparing nickel alumina catalysts with high efficiency and stability for dry reforming of methane consists of: (i) preparing a nanostructured mesoporous hybrid material within which nickel cations efficiently dispersed in contact with the alumina precursors, (ii) calcining the material to remove the organic part (linkers or structuring agents) and finally (iii) reducing at high temperature (above 700 °C) to form small and stable Ni^0^ nanoparticles anchored to the mesoporous alumina support. With such preparation route, the Ni^2+^ ions highly dispersed within the alumina after the step of calcination tend to form small and homogeneously distributed nickel aluminate (NiAl_2_O_4_) nanodomains that are embedded within the high specific surface area oxide matrix. Upon reduction, these NiAl_2_O_4_ nanospecies play a key role by boosting the metal-support interaction that persists even after Ni^0^ extraction towards the alumina surface, possibly because of the presence of a remaining mixed-metal phase at the metal-support interface. This stabilizes the supported Ni^0^ against sintering and protects the catalysts against development of carbon nanotubes usually formed on external weakly anchored Ni^0^ particles.

## Figures and Tables

**Table 1:** Properties and catalytic performance of the Ni_5wt%-_Al_2_O_3-MOF_ and Ni_5wt%-_Al_2_O_3-EISA_ materials.

Samples	Textural Properties of Calcined and Reduced *(in Italic)* Materials ^a^			Temperature of Reduction ^b^ (°C)	Ni° Nanoparticle ^c^ Size (nm)		Catalytic Results ^d^		
	S.A. (m^2^·g^−1^)	V_tot_ (cm^3^·g^−1^)	Ø (nm)		Reduced	Spent	Conversions (%)		H_2_/CO Molar Ratio
							X_CH4_	X_CO2_	
Ni_5wt%_-Al_2_O_3-MOF_	240 *(200)*	0.82 *(1.02)*	10 *(16)*	828	6.8	7.5	76	79	1.03
Ni_5wt%_-Al_2_O_3-EISA_	229 *(196)*	0.49 *(0.40)*	6.2 *(5.7)*	708	7	-	71	75	1.09

^a^: total surface area (S.A.), total pore volume (V_tot_) and mean pore diameter (Ø) calculated from the N_2_ sorption isotherms. ^b^: temperature at the maximum of TPR peaks. ^c^: mean sizes of Ni^0^ nanoparticles established from TEM histograms or from XRD patterns (in bracket). ^d^: performance in dry reforming of methane carried out at atmospheric pressure after 13 h at 650 °C and under a GHSV of 72 L·g^−1^·h^−1^.
